# Surgical Decision‐Making in the Setting of Diagnostic Uncertainty in Upper Extremity Subcutaneous Emphysema

**DOI:** 10.1155/cro/5229108

**Published:** 2026-07-22

**Authors:** Annie R. Waite, Sanjit Menon, Clayton W. Maschhoff, Paul P. Hospodar, Kaushik Bagchi

**Affiliations:** ^1^ Department of Orthopaedic Surgery, Albany Medical Center, Albany, New York, USA, amc.edu; ^2^ Albany Medical College, Albany, New York, USA, amc.edu

**Keywords:** benign subcutaneous emphysema, case report, necrotizing fasciitis, necrotizing soft tissue infection

## Abstract

**Background:**

Necrotizing soft tissue infections (NSTIs) are life‐threatening and require emergent surgical debridement. Benign subcutaneous emphysema is a rare, noninfectious condition characterized by subcutaneous gas on imaging without systemic symptoms or abnormal laboratory values typical of NSTIs. Patients with subcutaneous emphysema and risk factors for NSTI can pose a diagnostic challenge.

**Case Report:**

We report an 18‐year‐old female with Ehlers–Danlos syndrome who developed left elbow pain and crepitus following multiple insect bites, freshwater pond exposure, and contact with farm animals. She was afebrile with a benign exam and normal labs, yet imaging demonstrated extensive upper extremity subcutaneous emphysema. Given the concern for NSTI, she was admitted on broad‐spectrum antibiotics and underwent surgical exploration. Normal intraoperative findings, along with negative cultures and pathology, confirmed the diagnosis of benign subcutaneous emphysema. She was discharged postoperatively with oral antibiotics and made a full recovery.

**Conclusion:**

This case highlights the importance of multidisciplinary evaluation in differentiating benign from life‐threatening subcutaneous emphysema to guide appropriate management and avoid unnecessary surgery.

## 1. Introduction

Subcutaneous emphysema is defined as the presence of gas or air trapped under the skin and soft tissues, an alarming clinical observation that raises immediate concern for necrotizing soft tissue infections (NSTI) such as necrotizing fasciitis or gas gangrene [1]. Although uncommon, NSTIs are rapidly progressive, life‐threatening infections with high rates of morbidity and mortality, often requiring emergent surgical debridement [2]. However, benign subcutaneous emphysema is a rare, noninfectious condition that can present with subcutaneous gas on imaging without the constitutional symptoms or abnormal laboratory markers seen in NSTIs [3]. Documented cases of benign subcutaneous emphysema have occurred secondary to mild lacerations in the setting of trauma and insect bites [4–7]. These patients pose a diagnostic challenge for clinicians, as delays in treatment of NSTIs can result in increased morbidity and mortality, but reports of benign subcutaneous emphysema have been managed conservatively with prophylactic antibiotics and serial observation [4, 8–12]. We discuss the case of an otherwise healthy adolescent with Ehlers–Danlos syndrome (EDS) who developed extensive upper extremity subcutaneous emphysema after sustaining multiple insect bites with subsequent exposure to a freshwater pond and farm animals. This case is unique due to the rare combination of risk factors for subcutaneous emphysema, including EDS, minor trauma from insect bites, and freshwater exposure, and it highlights the importance of multidisciplinary decision‐making in distinguishing benign from life‐threatening etiologies of subcutaneous emphysema to avoid unnecessary surgical intervention in future cases.

This report follows the CARE guidelines to improve the quality of this case report.

## 2. Case Report

An 18‐year‐old, right‐hand dominant female with a past medical history of EDS presented to an urgent care with a 4‐day history of atraumatic pain and crepitus of her left elbow. She denied any recent penetrating trauma, but she reported a history of multiple mosquito bites to her left arm that she had been scratching frequently. Additionally, she had recently submerged her left arm into a freshwater pond at a horse barn and had exposure to farm animals while working as a veterinary technician. She denied any fever, chills, nausea, or vomiting. X‐rays at urgent care revealed subcutaneous emphysema concerning for a gas‐forming soft tissue infection (Figure 1). She was transferred urgently to the emergency department for surgical evaluation and a higher level of care.

**Figure 1:**
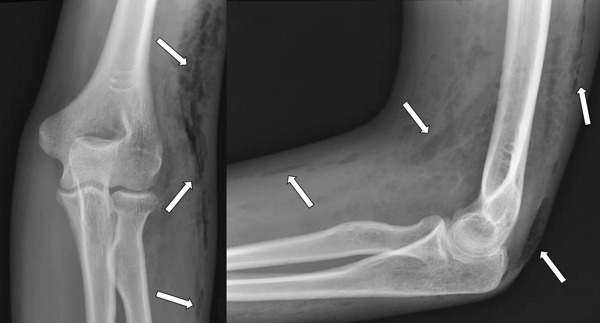
AP and lateral x‐ray of left elbow. Interpretation: Extensive lucencies within the soft tissues without osseous abnormalities, concerning for gas‐forming infection such as necrotizing soft tissue infection.

On examination by the orthopedic team, the patient was well‐appearing, afebrile, and hemodynamically stable. There were scattered insect bites about the left elbow, but there were no visible open wounds or lacerations. She had no erythema of the extremity. She was able to range the left wrist, elbow, and shoulder with minimal pain, but she reported tenderness around the elbow. Palpable crepitus was felt along the lateral aspect of the arm, extending from the wrist through the middle of the upper arm. A CT scan of the left upper extremity revealed extensive subcutaneous emphysema from the upper arm through the distal forearm without evidence of intramuscular or deep fascial extension (Figure 2). Laboratory values were unremarkable with a white blood cell (WBC) count of 4800 cells/*μ*L, erythrocyte sedimentation rate (ESR) of 11 mm/h, C‐reactive protein (CRP) of 1.8 mg/dL, and lactate of 0.8 mmol/L (Table 1).

**Figure 2:**
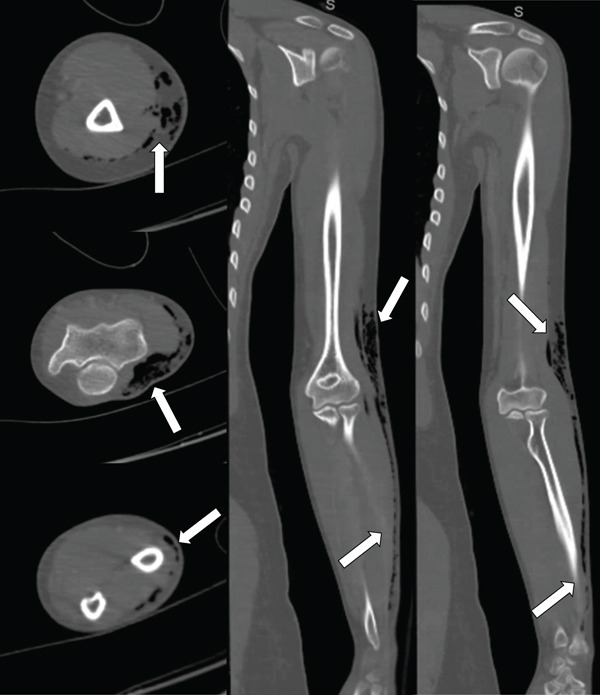
Axial and coronal cuts from a CT scan of the left upper extremity. Interpretation: extensive subcutaneous emphysema extending from the upper arm to the distal forearm without evidence of deep involvement of the fascia or muscle, concerning for a necrotizing soft tissue infection.

**Table 1:** The laboratory values obtained from the patient at the time of admission. Variables included in calculating an LRINEC score are noted.

Laboratory values		Normal	Included in LRINEC score
C‐Reactive protein (mg/L)	1.8	< 8.0	✔
Total white blood cell count (1000 cells/*μ*L)	4.8	4.8–10.8	✔
Hemoglobin(g/dL)	11.7	12.0–18.0	✔
Sodium (mmol/L)	128	128–145	✔
Creatinine(mg/dL)	1.0	0.6–1.2	✔
Glucose(mg/dL)	104	73–118	✔
Erythrocyte sedimentation rate (mm/h)	11	11–19	
Lactic acid (mmol/L)	0.8	0.4–2.0	

Despite her benign clinical picture, the extensive amount of subcutaneous emphysema seen on imaging in conjunction with her exposure to freshwater and farm animals was concerning for possible infection, so she was admitted and started on piperacillin–tazobactam and linezolid for broad‐spectrum antibiotic coverage with plans for frequent re‐evaluation. Her clinical exam remained unchanged the following morning. After a detailed discussion with the patient and her mother, the decision was made to proceed to the operating room for an exploration and incision and drainage given her significant risk factors.

Intraoperatively, two incisions were made, one along the proximal lateral forearm and another along the posterolateral upper arm, and dissection was carried down through the skin, subcutaneous tissue, fascia, and muscle. No purulence or fluid collections were noted. Fasciotomies were performed, and the muscles appeared contractile and healthy, with no evidence of necrosis. Intraoperative findings were normal, and there was no fascial or intramuscular involvement concerning for NSTI. Tissue specimens from muscle, fascia, and subcutaneous tissue were obtained and sent for microbiologic and pathologic examination. The wounds were copiously irrigated, closed with nonabsorbable sutures, and covered with dry sterile dressings.

She was seen by the infectious disease team postoperatively who suggested that the subcutaneous emphysema in combination with normal laboratory values, a benign clinical exam, and negative intraoperative findings could be suggestive of benign subcutaneous emphysema, a rare but noninfectious condition. They recommended continued coverage with ampicillin/sulbactam for 24 hours postoperatively, with a plan to transition to amoxicillin clavulanate at discharge. She was discharged the following day with a 3‐day course of antibiotics. Finalized cultures and pathology were negative.

At her 1‐week follow‐up visit, she demonstrated normal wound healing without crepitus or erythema. She reported only mild elbow pain and maintained full range of motion of the affected extremity. A timeline of events can be seen in Figure 3.

**Figure 3:**
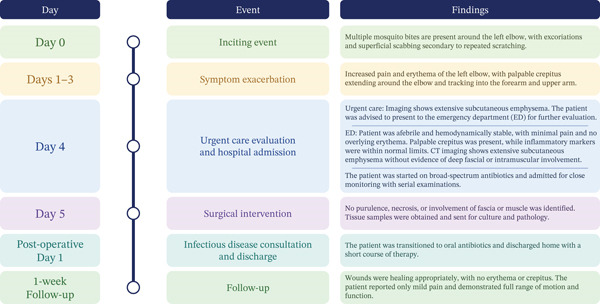
A timeline of events.

## 3. Discussion

Patients with subcutaneous gas on imaging without obvious clinical and laboratory signs of infection pose a diagnostic challenge for physicians. All cases of subcutaneous emphysema warrant a thorough workup for potential NSTIs to determine the need for emergent irrigation and debridement. Rarely, patients can present with benign subcutaneous emphysema, a noninfectious, self‐resolving condition [3–7, 9, 10, 12–14]. The purpose of this report is to highlight a case of benign subcutaneous emphysema with significant infectious risk factors and to analyze the decision‐making process for treatment.

NSTIs typically present with rapid progression of erythema, swelling, severe pain, and constitutional symptoms such as high fever, chills, and rigors [15]. Despite her benign clinical exam and lack of constitutional symptoms, the patient had several factors that increased her risk for infection. Exposure to a freshwater pond placed her at risk for *Aeromonas* species, whereas contact with farm animals and soil increased her risk for *Clostridium* species—both of which are associated with NSTIs [16, 17]. Insect bites and subsequent scratching also create a potential portal of entry for pathogens that can cause life‐threatening infections [18].

On the other hand, her clinical examination and laboratory findings were inconsistent with NSTI. The progression of her symptoms over 4 days without clinical deterioration is inconsistent with the typical course of NSTIs [15]. She was hemodynamically stable and afebrile with no erythema and minimal pain. Her laboratory values were all within normal ranges. The Laboratory Risk Indicator for Necrotizing Fasciitis (LRINEC) score, which uses six routine lab values to assess the risk for necrotizing fasciitis, was calculated at 1 which indicates a low risk of necrotizing fasciitis (Table 2) [19]. Despite the reassuring evidence, surgical exploration was deemed necessary given her significant risk factors.

**Table 2:** The variables included in the calculation for the Laboratory Risk Indicator for Necrotizing Fasciitis (LRINEC) score. This score is used to predict the probability that a patient has necrotizing fasciitis.

Variable	Value	Score
C‐reactive protein (mg/L)	≤ 150	0
	> 150	4

Total white blood cell count (1000 cells/*μ*L)	< 15	0
	15–25	1
	> 25	2

Hemoglobin (g/dL)	> 13.5	0
	11–13.5	1
	< 11	2

Sodium (mmol/L)	≥ 135	0
	< 135	2

Creatinine (mg/dL)	≤ 1.6	0
	> 1.6	2

Glucose (mg/dL)	≤ 180	0
	> 180	1

**Risk category**	**LRINEC points**	**Probability of necrotizing fasciitis**
Low	≤ 5	< 50%
Medium	6–7	50%–70%
High	≥ 8	> 75%

There is limited published literature on benign subcutaneous emphysema, and there are few documented cases secondary to insect bites [6, 7]. The proposed mechanism is the creation of a one‐way ball‐valve mechanism that traps air within the subcutaneous tissues [4]. In the setting of EDS, underlying dermal fragility may facilitate this process by permitting air entry through minor skin breaches, such as insect bites or superficial scratching [20]. Rare cases of facial or cervicothoracic subcutaneous emphysema and other air‐leak complications (e.g., pneumothorax and pneumomediastinum) have been described in EDS patients; however, to our knowledge, subcutaneous emphysema of the extremity following minor trauma has not been previously reported in this population, highlighting the uniqueness of this case and the associated diagnostic challenges in patients with underlying connective tissue disorders [21, 22].

Although some cases have been treated conservatively with observation, there are some patients who underwent surgical intervention due to concerns for NSTI [7, 13]. In this case, the decision to operate was guided by a prioritization of patient safety in the midst of diagnostic uncertainty and unestablished management guidelines. In retrospect, a trial of close observation may have been a reasonable alternative given her benign exam findings and low LRINEC score; however, after discussion with the surgical team and the patient, the potential risks of a missed NSTI outweighed the benefits of conservative management. Ultimately, normal intraoperative findings, along with negative cultures and pathology, confirmed the diagnosis of benign subcutaneous emphysema.

From an orthopedic perspective, the decision to proceed with surgical exploration was primarily driven by the extent of subcutaneous emphysema on imaging and the need to definitively exclude a limb‐threatening NSTI in the setting of significant risk factors. Although the patient was clinically stable with reassuring laboratory values, normal inflammatory markers do not eliminate concern for NSTIs when imaging demonstrates extensive emphysema, particularly in the setting of environmental contamination. In similar cases, surgeons should consider the distribution and severity of gas, including evidence of fascial or intramuscular involvement, as well as serial clinical examinations and progression of symptoms such as worsening pain, erythema, or systemic toxicity. When these factors raise concern, limited operative exploration may be warranted to establish a definitive diagnosis and avoid delaying treatment of a necrotizing infection.

This case emphasizes the importance of patient history and examination when imaging is inconsistent with the clinical picture. Clinical judgment should play a role in decision making, and in cases where there is no clinical or laboratory indication of infection, serial observation is an acceptable treatment. This case report serves to assist orthopedic surgeons in reevaluating the criteria for surgical intervention in patients who present with atypical subcutaneous emphysema.

## 4. Limitations

This case has several limitations. First, follow‐up was limited to 1 week, precluding assessment of longer‐term healing, recurrence, and functional outcomes following surgery. Second, the decision to operate is being evaluated retrospectively, and although surgical exploration was clinically justified at the time, close observation may also have been a reasonable alternative. Third, the LRINEC score may be less reliable in early or atypical NSTIs, and a low score should not be used to exclude NSTI but rather as an adjunct to clinical judgment and imaging. Finally, in the absence of established management guidelines for benign subcutaneous emphysema, treatment decisions must continue to rely on clinical assessment, imaging findings, and the overall patient risk profile.

## Funding

No funding was received for this manuscript.

## Consent

Informed consent was obtained from the patient for publication of this case report and any accompanying images.

## Conflicts of Interest

The authors declare no conflicts of interest.

## Supporting information


**Supporting Information** Additional supporting information can be found online in the Supporting Information section. CARE Checklist was used to follow CARE guidelines in manuscript preparation to improve quality of this case report.

## Data Availability

The data that support the findings of this study are available from the corresponding author upon reasonable request.
