# Supplementary figures and images for: Surgical Decision‐Making in the Setting of Diagnostic Uncertainty in Upper Extremity Subcutaneous Emphysema
Source: Case Rep Orthop. 2026 Jul 22;2026:5229108. doi: 10.1155/cro/5229108 (PMC13392414; doi:10.1155/cro/5229108)

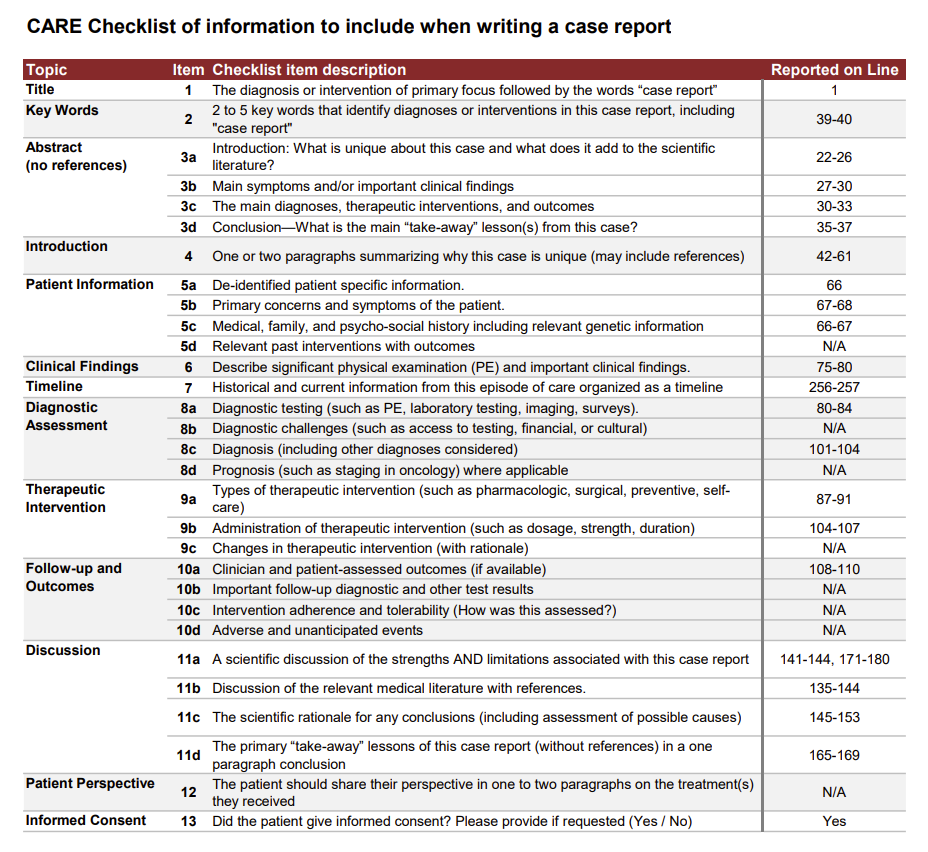

Supplement: Supplementary file 1 — Supporting Information Additional supporting information can be found online in the Supporting Information section. CARE Checklist was used to follow CARE guidelines in manuscript preparation to improve quality of this case report. [file CRO-2026-5229108-s001.docx]
